# Helminth-Based Product and the Microbiome of Mice with Lupus

**DOI:** 10.1128/mSystems.00160-18

**Published:** 2019-02-19

**Authors:** Hadar Neuman, Hadar Mor, Tomer Bashi, Or Givol, Abdulla Watad, Asaf Shemer, Alexander Volkov, Iris Barshack, Mati Fridkin, Miri Blank, Yehuda Shoenfeld, Omry Koren

**Affiliations:** a Azrieli Faculty of Medicine, Bar Ilan University, Safed, Israel; b Zabludowicz Center for Autoimmune Diseases, Tel-Hashomer, affiliated to Sackler Faculty of Medicine, Tel-Aviv University, Tel Aviv, Israel; c Institute of Pathology, Sheba Medical Center Sheba Medical Center, Tel-Hashomer, affiliated to Sackler Faculty of Medicine, Tel-Aviv University, Tel Aviv, Israel; d St. Petersburg University, Saint Petersburg, Russia; e Department of Organic Chemistry, The Weizmann Institute of Sciences, Rehovot, Israel; University of California, Riverside

**Keywords:** helminth, lupus, microbiome

## Abstract

The hygiene hypothesis claims that the lack of exposure to microorganisms in developed countries correlates with a rise in the incidence of autoimmune diseases. It was also found that helminths are able to modulate the immune response in hosts in order to survive. Consequently, several successful trials using helminths as a treatment for autoimmune patients have been reported. The helminth derivative, phosphorylcholine (PC), was discovered as an immunomodulatory molecule. We have recently shown in a murine model that when a conjugate of tuftsin and PC, termed TPC, is prophylactically administered before the onset of glomerulonephritis, it attenuates the development of systemic lupus erythematosus (SLE). The current study aimed to examine the TPC effect on the gut microbiome in a mouse model of lupus. TPC treatment altered the gut composition in the mice with active lupus, in correlation with a significant decrease in glomerulonephritis, followed by an increased level of anti-inflammatory interleukin 10 (IL-10), decreased levels of proinflammatory mediators, and expansion of the T regulatory cell population. Importantly, we found that TPC treatment altered the mouse gut microbiome composition, in correlation with a significant decrease in protein secretion and improved disease parameters. The major effects of TPC treatment on the gut microbiome included decreased abundances of *Akkermansia* and increased abundance of several genera, including *Turicibacter, Bifidobacterium*, unclassified *Mogibacteriaceae*, unclassified *Clostridiaceae*, *Adlercreutzia*, *Allobaculum*, and *Anaeroplasma*. Overall, our results associate microbial changes with the immunomodulation of glomerulonephritis in mice with lupus.

**IMPORTANCE** Recently, several papers referred to the association of different bacteria with lupus in mice and humans. This is the first report to demonstrate the effect of a compound derived from helminths on the induction of remission in mice with lupus and its association with a bacterial change. We show that several genera, including *Akkermansia*, are associated with clinical and serological parameters of lupus, while other genera, including butyrate-producing bacteria, are associated with amelioration of disease following tuftsin and phosphorylcholine treatment.

## Abstract (precis)

Recently, several papers referred to the association of different bacteria with lupus in mice and humans. This is the first report to demonstrate the effect of a compound derived from helminths on the induction of remission in mice with lupus and its association with a bacterial change. We show that several genera, including *Akkermansia*, are associated with clinical and serological parameters of lupus, while other genera, including butyrate-producing bacteria, are associated with amelioration of disease following tuftsin and phosphorylcholine treatment.

## INTRODUCTION

Systemic lupus erythematosus (SLE) is a chronic autoimmune disease which most often affects women and may involve diverse organs and systems (1). The pathogenesis of SLE involves a complex interplay between genetic predisposition, hormonal, and environmental factors (2). SLE treatment remains unsatisfactory, and despite the most recent innovative agents or ongoing trials, there is no generally effective approach for the treatment of this disease (3). Conventional immunosuppressive agents such as cyclophosphamide, mycophenolate mofetil, and azathioprine are extensively used in the management of SLE. Systemic corticosteroids remain a major therapy for SLE, but their long-term use is associated with adverse effects in diverse organs and systems (3, 4), at times requiring cessation of treatment (5). Consequently, treatment optimization while minimizing potential side effects remains essential (6). This requires a better understanding of the pathways involved in the disease and those affected by potential treatments.

A link between hygiene, sanitation, and the prevalence of autoimmune diseases has been established (7, 8). Microbial agents, particularly parasites, were demonstrated to exhibit a protective effect toward diverse autoimmune diseases through their immunomodulatory properties (9–11). Several studies have been conducted to evaluate the efficacy of parasites and helminth derivatives as potential treatments for a heterogeneous group of autoimmune diseases (12, 13). Helminths might be associated with differential changes in the levels of Th2 cytokines, with upregulation of regulatory interleukins, such as IL-4 and IL-10, and a downregulation of effector molecules, such as IL-5 and IL-13. Therefore, in spite of an overall “Th2 signature,” modulatory effects would prevail on stimulatory ones (14).

We have previously reported development of a synthetic tuftsin-phosphorylcholine (TPC) adduct, which may mimic the modulatory effect induced by parasites on the immune system (15, 16). Tuftsin is a tetrapeptide located in the Fc domain of the heavy chain of immunoglobulin G, which also has an immunostimulatory effect, and PC is an immunomodulatory molecule secreted by helminths (17). We showed that TPC can prevent dextransulfate sodium salt (DSS)-induced colitis in a murine model (15), resulting in reduced intestinal bleeding and weight loss, improved histology, and prolonged survival. TPC was additionally proven to exhibit therapeutic benefits in collagen-induced arthritis (CIA), leading to lower arthritic scores and healthy joint histology (17). Moreover, we showed that TPC can modulate the progression of established murine lupus nephritis and has a stimulatory effect on defective monocyte chemotaxis in SLE (16).

In recent years, there has been a growing recognition that the microbiome plays important roles affecting the host immune system, metabolism, and overall health. Gut microbiome compositions have been shown to be altered in a variety of autoimmune diseases, including inflammatory bowel disease (IBD), type 1 diabetes (T1D), multiple sclerosis (MS), and rheumatoid arthritis (RA) (18). In studies of both human SLE and murine disease models, the microbial compositions have been shown to be different from those of healthy controls (19). While a lower *Firmicutes*/*Bacteroidetes* ratio in SLE patients versus controls has been shown (20), more specific alterations have been found in SLE murine models, including a decrease in *Lactobacilli* abundance and an increase in *Lachnospiraceae* (21). The microbial alterations found in SLE may not be surprising as there is dual interplay between the microbiome and immune system, with bacterial composition shaping the immune system and affecting cytokine levels on the one hand and immune deficiencies affecting the microbial populations on the other hand (22). We therefore hypothesized that an efficient treatment for SLE, such as TPC, may also affect the microbiome.

In this study, we evaluated the therapeutic effects of TPC treatment administered at disease onset in mice with lupus and analyzed the effects of TPC on the gut microbiome. We show beneficial effects of TPC treatment in mice with established SLE, including an increase in the level of anti-inflammatory cytokines, a decrease in the levels of proinflammatory mediators, and expansion of the Treg cell population. We additionally demonstrate effects of the treatment on gut microbial composition, in correlation with a significant decrease in urinary protein and improved disease parameters. Overall, our results show that both immune and gut microbiome parameters are significantly altered by TPC treatment in a murine SLE model, in correlation with mitigation of disease.

## RESULTS

In the current study, we analyzed the effect of TPC on disease parameters and the gut microbiome in mice with established lupus nephritis.

### Improvement of glomerulonephritis upon TPC treatment in mice with active lupus.

**FIG 1:**
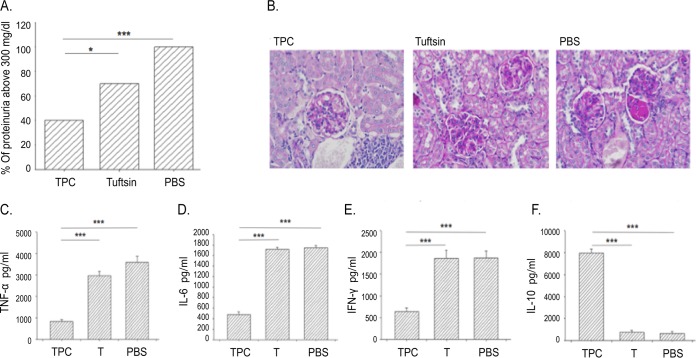
TPC attenuates glomerulonephritis in treated lupus-prone mice. (A) Proteinuria. The average levels of proteinuria are presented as percentages of mice that had greater than 300 mg/dl protein, in each group of mice (*n* = 15 per group) treated with tuftsin-phosphorylcholine (TPC), tuftsin (T), or PBS (vehicle). (B) Histological analysis. PAS staining in representative kidney sections from each group of mice studied. Magnification, ×200. Concentrations of the proinflammatory cytokines TNF-α (C), IL-6 (D), IFN-γ (E), and the anti-inflammatory cytokine IL-10 (F) secreted in the culture fluids of splenocytes originating from TPC-, tuftsin (T)-, and PBS-treated mice. The data are presented as concentrations in pg/ml; *n* = 10 per group. *, *P* < 0.02; ***, *P* < 0.001.

Female NZBxW/F1 lupus-prone mice were subcutaneously injected with TPC, tuftsin, or phosphate-buffered saline (PBS) starting at week 26 after the clinical onset of SLE (*n* = 15 per group). As shown in Fig. 1A, at the age of 32 weeks, 100% of the mice injected with PBS developed severe proteinuria (defined as ≥300 mg/dl), whereas only 40% of the TPC-treated group exhibited proteinuria above 300 mg/dl. TPC treatment in mice significantly prevented deterioration to high levels of proteinuria compared to that in control PBS- or tuftsin-treated mice (*P* < 0.001 or *P* < 0.02, respectively). Moreover, at 35 weeks, the mice were sacrificed, and histological analyses of the kidneys by periodic acid-Schiff base (PAS) staining was performed (*N* = 15/group). The renal parenchyma of TPC-treated mice exhibited mild mesangial cell proliferation with mild mesangial matrix widening (13/15 mice showed mild symptoms, while 2/15 showed focal proliferative glomerulonephritis). Kidney tissue from tuftsin-treated mice showed focal proliferative glomerulonephritis (involving less than 50% of glomeruli) in 7/15 cases. Kidney tissue from PBS-treated mice presented diffuse proliferative glomerulonephritis with formation of multiple crescents in 13/15 cases (Fig. 1B).

### Effects of TPC on cytokine profiles.

Mice with lupus were treated with TPC, tuftsin, or PBS. The cytokine secretion from isolated splenocytes was analyzed *ex vivo*. Splenocytes derived from mice with lupus treated with TPC showed significantly decreased secretion of proinflammatory cytokines (tumor necrosis factor alpha [TNF-α], gamma interferon [IFN-γ], and IL-6) and enhanced production of IL-10 compared to those from mice treated with tuftsin and PBS (*P* < 0.001) (Fig. 1C to F). The concentration of TNF-α in comparison to that from splenocytes derived from PBS-treated mice decreased by 4.25-fold, IFN-γ by 3-fold, and IL-6 by 3.6-fold, whereas mice with lupus treated with TPC exhibited a 3.5-fold reduction of proinflammatory cytokine TNF-α secretion compared to that from mice with lupus treated with tuftsin.

An analysis of anti-inflammatory cytokine IL-10 levels in the culture fluid of splenocytes derived from mice with lupus treated with TPC revealed increased IL-10 secretion by 12.5-fold compared to that from splenocytes originating from PBS-treated mice (*P* < 0.001).

**FIG 2:**
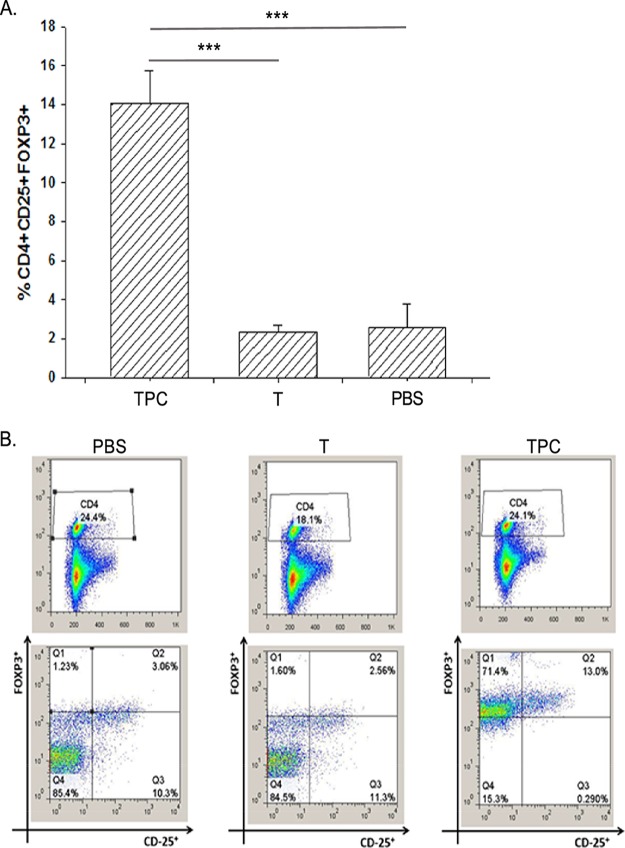
TPC expands the number of Tregs in mice treated after disease onset. (A) Percentages of Treg expansion in the spleens of TPC-, tuftsin (T)-, and PBS-treated mice. The data are presented as means ± SDs (*n* = 15 for each group). (B) Representative FACS analyses of CD4^+^CD25^+^FOXP3^+^ splenocytes derived from the TPC-, tuftsin (T)-, and PBS-treated mice.

TPC increased the number of regulatory T cells (Tregs) in mice with established lupus. The frequency of CD4^+^/CD25^+^/FOXP3^+^ Treg subsets was determined *in vitro* in the splenocytes culture fluid following treatment with TPC, tuftsin, and PBS (Fig. 2A and B). A significant increase (*P* < 0.001) in the percentage of Tregs was observed in the TPC-treated group compared with those in animals treated with tuftsin or PBS (14%, 2.34%, and 2.58%, respectively) following a second *in vitro* boost of 1 μg/ml. Treg levels in splenocytes derived from TPC-, tuftsin-, and PBS-treated mice are presented in Fig. 2B.

### TPC treatment affects the gut microbiome.

**FIG 3:**
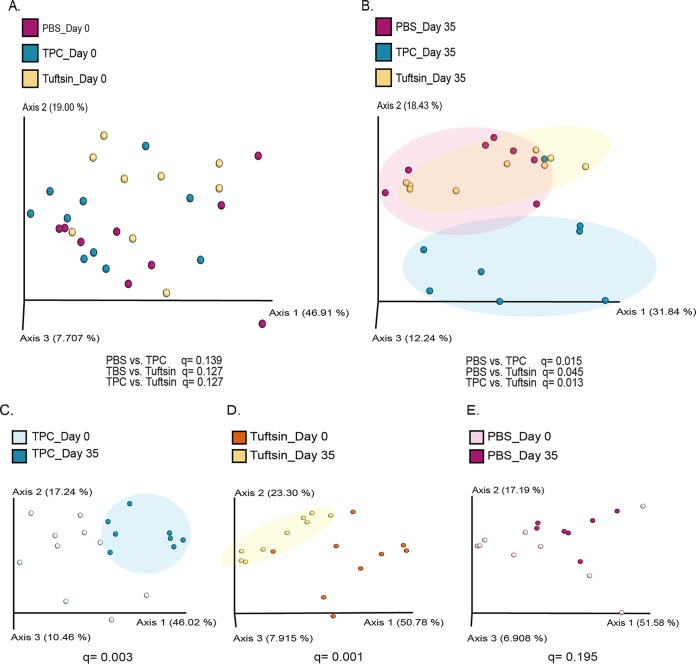
Microbial communities of the differently treated mice with lupus cluster separately after treatment. Samples were clustered using a PCoA of weighted UniFrac distance matrix (*N* = 7 to 10/group) at day 0 (A), day 35 (B), day 0 versus 35 of TPC-treated mice (C), day 0 versus day 35 in tuftsin-treated mice (D), and day 0 versus day 35 in PBS-treated mice (E).

**FIG 4:**
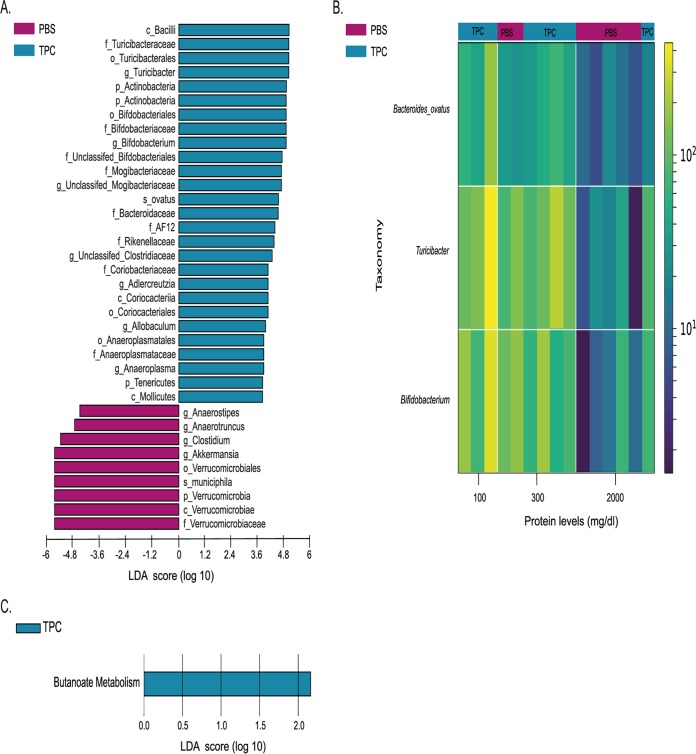
Microbial differences found between TPC- and PBS-treated mice with lupus. (A) Bar chart of significant differences in gut bacterial abundances between TPC- and PBS-treated mice with lupus at day 35 based on LEfSe analysis. (B) Heatmap of differential features correlated with protein levels in TPC- versus PBS-treated mice with lupus at day 35, generated using Calour. Bacteria were selected using Pearson's correlation with a dsFDR of 0.05 multiple hypothesis correction. Top color bars (TPC/PBS) indicate the different treatments. Side color bar indicates relative reads out of 10,000 (following normalization of each sample to 10,000 reads). (C) Bar chart of significant differences in predicted gut bacterial function between TPC- and PBS-treated mice with lupus at day 35 based on PICRUSt analysis.

To test the effects of TPC and tuftsin on the microbiome, we tested the microbial compositions in fecal samples from mice receiving each of the three treatments at day 0 and at day 35 of treatment by next-generation sequencing (NGS) of the 16S rRNA gene. Principal-coordinate analysis (PCoA) was performed based on a weighted UniFrac distance matrix. At the starting point of the experiment, the microbial communities of all treatment groups were similar, as seen by their clustering at day 0 (Fig. 3A; see also Fig. S1A in the supplemental material). However, after 35 days of treatment, the TPC-treated mice with lupus had a unique microbial profile, as seen by separate clustering from tuftsin- and PBS-treated mice with lupus (Fig. 3B; Fig. S1B). Interestingly, while the microbial populations of the TPC-treated mice appeared most different from the PBS-treated samples, the tuftsin-treated group had an intermediate composition. When comparing microbial profiles of each treatment group at day 35 versus day 0, both TPC and tuftsin treatments showed significant differences between time points (Fig. 3C and D), while the PBS treatment did not (Fig. 3E). Additionally, we found significant differences in gut microbial compositions following TPC versus PBS treatments, as evident by differences in the abundances of bacterial communities at day 35 (Fig. 4). TPC-treated mice had lower relative abundances of Akkermansia muciniphila (linear discriminant analysis score [LDA] = 5.17, *P* = 0.002) and the entire *Verrucomicrobia* phylum (LDA = 5.17, *P* = 0.002), as well as the genera *Clostridium, Anaerostipes*, and *Anaerotruncus*, than PBS-treated mice. On the other hand, TPC-treated mice exhibited higher relative abundances of the genera *Bifidobacterium*, *Turicibacter*, unclassified *Mogibacteriaceae*, unclassified *Clostridiaceae*, *Adlercreutzia*, *Allobaculum*, and *Anaeroplasma*, as well as the species Bacteroides ovatus and the families AF12 and *Rikenellaceae*, than PBS-treated mice (Fig. 4A). The significant increase in abundance of Akkermansia muciniphila in the TPC versus PBS treatment groups was also evident by analysis of composition of microbes (ANCOM) (see Fig. S2).

10.1128/mSystems.00160-18.1 FIG S1 Microbial communities of TPC- versus PBS-treated mice with lupus. Samples were clustered using a PCoA of weighted UniFrac distance matrix. (A) Day 0. (B) Day 35. *q* values show significant differences between groups at day 35 (*q* = 0.004) but not at day 0 (*q* = 0.174). Download FIG S1, PDF file, 0.2 MB. Copyright © 2019 Neuman et al. 2019 Neuman et al. This content is distributed under the terms of the Creative Commons Attribution 4.0 International license.

10.1128/mSystems.00160-18.2 FIG S2 The abundance of Akkermansia muciniphila in TPC- versus PBS-treated mice with lupus at day 35. Relative abundance according to ANCOM analysis is presented as boxplots (W = 21, FDR = 0.005). Download FIG S2, PDF file, 0.02 MB. Copyright © 2019 Neuman et al. 2019 Neuman et al. This content is distributed under the terms of the Creative Commons Attribution 4.0 International license.

Additionally, we correlated the taxonomical feature abundances at day 35 between the TPC- and PBS-treated mice with the protein levels per mouse, as shown in the heatmap presented in Fig. 4B. We found the abundance of Bacteroides ovatus, as well as *Turicibacter* and *Bifidobacterium* genera, negatively correlated with high protein levels. Generally, PBS-treated mice had higher levels of protein in the urine (5/7 versus 1/8 TPC-treated mice displayed 2,000 mg/dl) and lower abundances of Bacteroides ovatus, *Turicibacter*, and *Bifidobacterium* than TPC-treated mice. TPC-treated mice exhibited low protein levels in the urine (3/8 mice displayed 100 mg/dl, 4/8 mice displayed 300 mg/dl).

To characterize the different functional alterations in the gut microbiota of TPC- versus PBS-treated mice, we predicted the functional composition profiles using 16S rRNA sequencing data analyzed with PICRUSt. Of the 328 KEGG (level 3) pathways tested, we found butanoate metabolism to be significantly more abundant in TPC-treated mice than in PBS-treated mice at day 35 (LDA = 2.16; *P* = 0.007) (Fig. 4C). Overall, significant effects of TPC treatment on the gut microbiota compositions were observed.

## DISCUSSION

Previously, we demonstrated the favorable effect of TPC in the modulation and prevention of nephritis in lupus-prone mice (16). In the current study, we examined the effect of TPC in lupus-prone mice when starting the administration at 26 weeks after disease onset. In this model, TPC significantly reduced the proteinuria levels in comparison with those in mice treated with PBS. We observed a significant decrease of the proinflammatory cytokines, including TNF-α, IL-6, and IFN-γ, and a significant increase in anti-inflammatory cytokines such as IL-10. Moreover, TPC caused an expansion in the number of CD4^+^CD25^+^FOXP3^+^ Treg phenotype cells.

A recent study aimed to evaluate the effect of a different helminth derivative, a glycoprotein produced by the filarial nematode Acanthocheilonema viteae, named ES-62. This compound was reported to suppress plasmablast differentiation and to thereby attenuate the autoantibody production and immune complex deposition in the kidney in SLE. Only 22% of mice treated with ES-62 developed proteinuria, while proteinuria developed in 100% in those treated with PBS (23). The authors initiated the treatment with ES-62 at 21 weeks, while in our study, treatment was initiated at week 26; this difference may explain the higher percentage of proteinuria-positive animals among the TPC-treated mice in comparison to those treated with ES-62. In our study, the administration of TPC had a favorable impact on glomerulonephritis in terms of histological findings, as both TPC and tuftsin treatment led to a reduction in immune complex deposition in the mesangium, with an even greater effect in the TPC-treated group of mice.

We found that TPC significantly alters the microbiome composition of TPC-treated mice with lupus, including changes correlated with decreased protein levels in the urine. In TPC-treated mice, there was a significant decrease in Akkermansia muciniphila abundance, as well as for the genera *Clostridium*, *Anaerostipes*, and *Anaerotruncus*. A. muciniphila was shown to exacerbate gut inflammation, and a high abundance of this species has previously been correlated to DSS-induced colitis in mice (24, 25). It was also found that A. muciniphila activates the NF-κB pathway through Toll-like receptor 4 (TLR4) and TLR2. A. muciniphila lipopolysaccharide (LPS), which is most likely the activating molecule for TLR2 and TLR4, induced production of IL-8, IL-6, and small amounts of IL-10 and TNF-α in PBMCs. Purified recombinant Amuc_1100, an outer membrane pilus-like protein, specifically induces TLR2 and is able to induce IL-1β, IL-6, IL-8, IL-10, and TNF-α production in peripheral blood mononuclear cells (PBMCs) as well. In another study, A. muciniphila-derived extracellular vesicles (EV) secreted increasing amounts of IL-6 in a dose-dependent manner (26). The *Anaerotruncus* genus has also been previously associated with disease, as its abundance is elevated in colorectal cancer patients versus that in healthy controls (27).

On the other hand, TPC-treated mice exhibited higher abundances of several genera, among these, *Bifidobacterium* and *Adlercreutzia*. These genera may protect the mice from disease deterioration. In agreement with this, *Adlercreutzia* was previously shown to be decreased in children with IBD (28). Moreover, *Bifidobacterium* is a widely used probiotic with proven positive effects in numerous disease states (29). These effects are attributed to short-chain fatty acid (SCFA) production, especially lactate production, which is further metabolized to butyrate. This fits our finding that the butyrate metabolism pathway is more expressed in TPC-treated than in PBS-treated mice. Butyrate plays protective roles in maintaining the mucus layer of the intestinal barrier, mainly via gene transcription (e.g., increasing expression of MUC2, PGE1, etc.) (28, 30).

Altogether, these results present the microbiome as an important and novel factor that may mediate TPC treatment, immune changes, and improvement in glomerulonephritis parameters. Since SLE is associated with microbial dysbiosis, it is not surprising that an effective SLE treatment positively affects the microbiome by promoting beneficial populations. Further linking of these components may improve our understanding of disease etiology and optimize treatments. These results further suggest testing the use of a *Bifidobacterium* probiotic as a dietary supplement to relieve kidney injury in lupus patients.

In conclusion, our study shows that TPC has immunomodulatory effects in active SLE when administered postonset. TPC was found to substantially reduce the levels of proteinuria through diverse mechanisms, by which proinflammatory cytokines were reduced and anti-inflammatory cytokines were increased, as well as clear expansion of CD4^+^CD25^+^FOXP3^+^ Treg cells. Likewise, the immunomodulatory activity of TPC was associated with changes in the gut microbiome composition, including both elevation of beneficial bacterial populations and reduction of bacteria that may promote inflammation. These results highlight the gut microbiome as another mechanism that may explain the immunomodulatory activity of TPC in mice with lupus.

## MATERIALS AND METHODS

### Tuftsin-phosphorylcholine.

Tuftsin is a synthetic phagocytosis-stimulating tetrapeptide (Thr-Lys-Pro-Arg) produced by enzymatic cleavage in the spleen (31, 32). This natural tetrapeptide entails several biological activities and effects which are related to the immune system (33). Tuftsin was elongated by addition of Gly-Tyr, to which PC was conjugated by azo bond to construct TPC.

### Mice and experimental design.

All studies were performed using female SLE-prone (NZBxW) F1 mice (14 to 15 weeks old; Envigo, UK), an established model for SLE nephritis. The mice were fed and maintained in the animal house of the Sheba Medical center. Mice were separated into three treatment groups: TPC treated, Tuftsin treated, and PBS treated. TPC, tuftsin, or PBS was administered subcutaneously at a dose of 5 μg/per mouse 3 times a week starting after the establishment of nephritis determined by the documentation of proteinuria (100 mg/dl). The experiment was approved under the protocols of the ethics committee of the Israeli Ministry of Health (number 696/11).

### Proteinuria.

To establish proteinuria, mouse urine was tested weekly with a standard semiquantitative test, using dipsticks (Multistix; Bayer, Fernwald, Germany), according to the manufacturer's instructions. The presence of 100 to 2,000 mg/dl protein in the urine was defined as nephritis.

### Histologic analysis of glomerulonephritis.

To detect histological signs of glomerulonephritis, kidneys were collected from all mice sacrificed by cervical dislocation and were paraffin embedded (*N* = 45). Sections were stained with periodic acid-Schiff base (PAS) and examined microscopically by a certified histopathologist.

### Cytokine measurements.

Spleen cells were derived from mice treated with TPC, tuftsin, and PBS. Cells were pressed though a sterile strainer into RPMI 1640 medium containing 1% fetal calf serum (FCS; Beit HaEmek, Israel). After washing the cells in serum-free RPMI 1640, erythrocytes were lysed in red-blood-cell lysis buffer (Beit HaEmek, Israel), and cell viability was determined by trypan blue exclusion. Cells were cultured as single-cell suspensions, 5 × 10^5^ cells/well in 24-well plates coated with anti-CD3 antibody (Ab; 2 μg/ml) in the presence or absence of 5 μg/ml TPC, tuftsin, or PC for 72 h in enriched medium at 37°C in 5% CO_2_. Culture supernatants were collected at various times. The concentrations of the cytokines in the culture fluid were tested by Duoset (R&D systems, Minneapolis, MN, USA) sandwich ELISA according to the manufacturer's instructions.

### T regulatory cell analysis by flow cytometry.

After the mice were sacrificed, their spleens were harvested. Initially, spleen cells were depleted of red blood cells. Next, isolated splenocytes were incubated with the relevant antibodies: anti-CD4-fluorescein isothiocyanate (FITC), anti-CD25, allophycocyanin (APC), and anti-FOXP3-phycoerythrin (PE) (eBioscience, San Diego, USA). Two microliters of anti-CD4-FITC (diluted 1:5 in PBS) and 2 µl of anti-CD25-APC (diluted 1:5 in PBS) were mixed with each of the 3 × 10^6^ splenocytes samples, which were incubated for 1.5 h at room temperature. The cells were then incubated overnight at 4°C with fixation solution, followed by suspension in permeabilization solution (Serotec, Oxford, UK). Splenocytes were then stained for FOXP3 by incubating them with anti-FOXP3-PE (diluted 1:5 in PBS) for 1.5 h at room temperature (16). Splenocytes were analyzed by fluorescence activated cell sorting (FACS), with forward and side scatter gates adjusted to include all cells and to exclude debris (Becton, Dickinson, Franklin Lakes, NJ, USA). The gating was performed on the CD4^+^ T cells.

### Gut microbiome analysis.

Fecal samples were collected at days 0 and 35 of treatment from TPC-, tuftsin-, and PBS-treated mice and kept at −80°C until analysis. DNA was extracted from feces using the Power Soil DNA isolation kit (Mo Bio) according to the manufacturer's instructions, using a Beadbeater (BioSpec) for 2 min. Following DNA extraction, the bacterial 16S rRNA gene V4 variable region was amplified, purified, quantified, and sequenced using an Illumina MiSeq platform as previously described (34). Data analysis was performed using QIIME2 (35). Sequence reads were demultiplexed by per-sample barcodes, and Illumina-sequenced amplicon read errors were corrected using DADA2 (36). A phylogenetic tree was generated and taxonomy classification was performed using the Greengenes reference database (37). Beta diversity were calculated based on a feature table containing features observed in at least 3 samples and on samples containing at least 11,000 sequences and then analyzed using principal coordinate analysis (PCoA) based on the weighted UniFrac distance matrices (38). We also performed linear discriminant analysis effect size (LEfSe) (39), which determines the features that are significantly different between samples according to relative abundances. Metagenome functional predictive analysis was carried out using PICRUSt (40). Analysis of composition of microbes (ANCOM) was used to identify features that are differentially abundant between sample groups (41). A heatmap was generated using EZCalour (https://github.com/amnona/EZCalour).

### Statistical analysis.

Statistical analysis was performed by unpaired Student's *t* tests and nonparametric Mann-Whitney tests. Results are expressed as means ± standard deviations (SDs). A *P* value of *<*0.05 was considered significant. Differences between weighted UniFrac distances were analyzed by pairwise permutational multivariate analysis of variance (PERMANOVA). Analysis of composition of microbiomes (ANCOM) was used at a *P* value cutoff of 0.05 and Benjamini-Hochberg false discovery rate (FDR) correction. LEfSe identified the significantly different features (*P *< 0.05) and corrected them for multiple comparisons using the Benjamini-Hochberg false discovery rate (FDR) correction. Calour used permutation-based correlation *P* values with multiple hypothesis correction at a discrete FDR (dsFDR) of 0.05 (42).

### Data availability.

The 16S rRNA gene sequence data have been deposited in the European Bioinformatics Institute (EBI) database with accession code ERP106749.
