# Supplementary figures and images for: Helminth-Based Product and the Microbiome of Mice with Lupus
Source: mSystems. 2019 Feb 19;4(1):e00160-18. doi: 10.1128/mSystems.00160-18 (PMC6381224; doi:10.1128/mSystems.00160-18)

Sup. Figure 1.

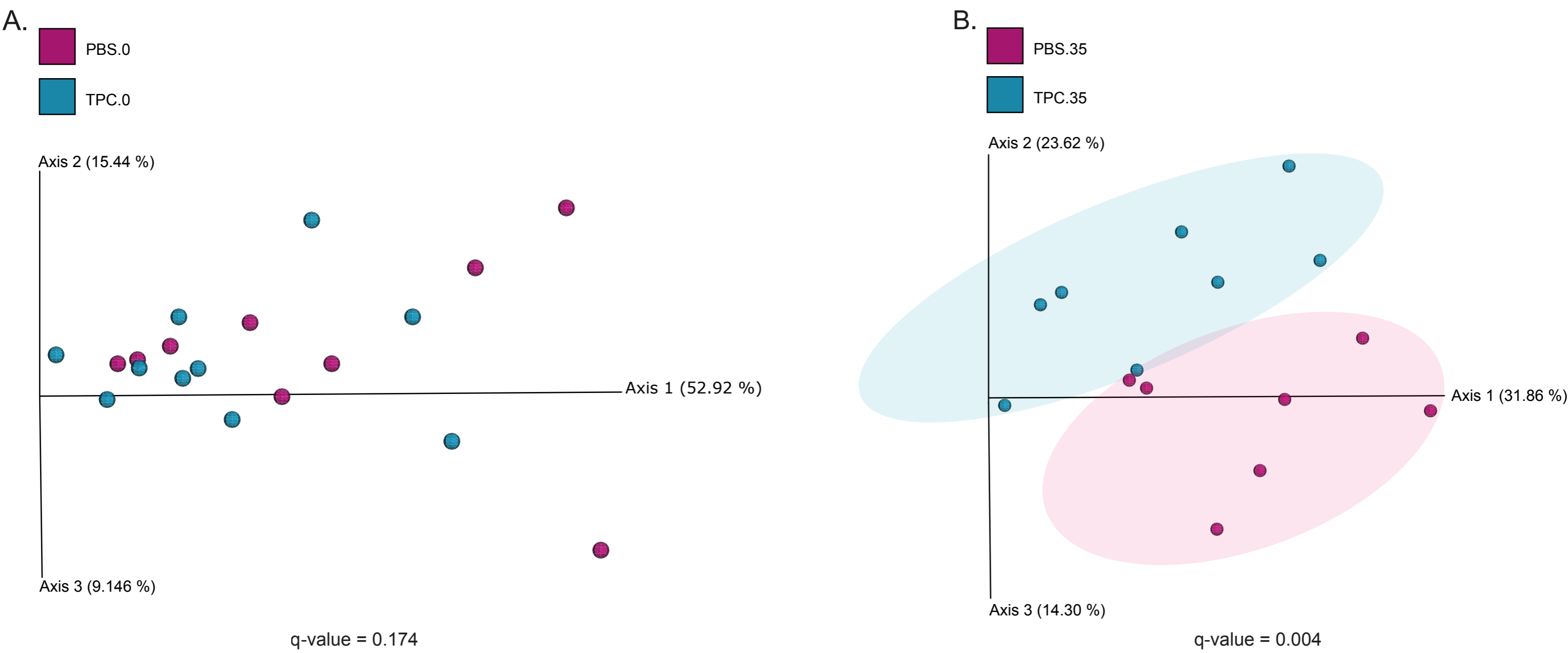

Fig.4

Supplement: FIG S1 [file mSystems.00160-18-sf001.pdf]

Sup. Figure 2.

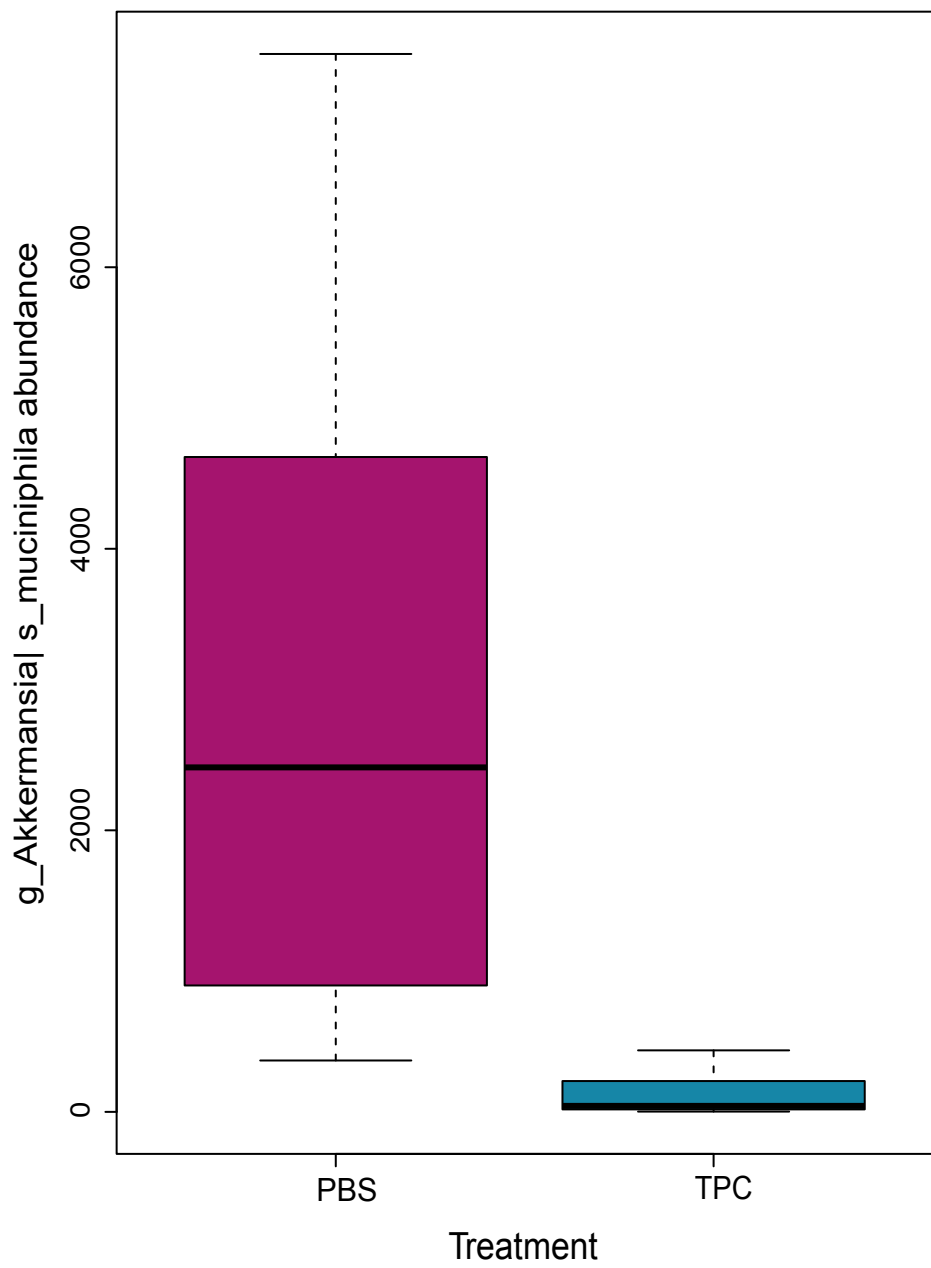

Supplement: FIG S2 [file mSystems.00160-18-sf002.pdf]
